# Flavored Tobacco Product Use Among Middle and High School Students — United States, 2014–2018

**DOI:** 10.15585/mmwr.mm6839a2

**Published:** 2019-10-04

**Authors:** Karen A. Cullen, Sherry T. Liu, Jennifer K. Bernat, Wendy I. Slavit, Michael A. Tynan, Brian A. King, Linda J. Neff

**Affiliations:** ^1^Center for Tobacco Products, Food and Drug Administration, Silver Spring, Maryland; ^2^Office on Smoking and Health, National Center for Chronic Disease Prevention and Health Promotion, CDC.

The 2009 Family Smoking Prevention and Tobacco Control Act prohibits the inclusion of characterizing flavors (e.g., candy or fruit) other than tobacco and menthol in cigarettes; however, characterizing flavors are not currently prohibited in other tobacco products at the federal level.* Flavored tobacco products can appeal to youths and young adults and influence initiation and establishment of tobacco-use patterns (*1*). The Food and Drug Administration (FDA) and CDC analyzed data from the 2014–2018 National Youth Tobacco Surveys (NYTS) to determine prevalence of current (past 30-day) use of flavored tobacco products, including electronic cigarettes (e-cigarettes), hookah tobacco, cigars, pipe tobacco, smokeless tobacco, bidis, and menthol cigarettes among U.S. middle school (grades 6–8) and high school (grades 9–12) students. In 2018, an estimated 3.15 million (64.1%) youth tobacco product users currently used one or more flavored tobacco products, compared with 3.26 million (70.0%) in 2014. Despite this overall decrease in use of flavored tobacco products, current use of flavored e-cigarettes increased among high school students during 2014–2018; among middle school students, current use of flavored e-cigarettes increased during 2015–2018, following a decrease during 2014–2015. During 2014–2018, current use of flavored hookah tobacco decreased among middle and high school students; current use of flavored smokeless tobacco, cigars, pipe tobacco, and menthol cigarettes decreased among high school students. Full implementation of comprehensive tobacco prevention and control strategies, coupled with regulation of tobacco products by FDA, can help prevent and reduce use of tobacco products, including flavored tobacco products, among U.S. youths (*2*,*3*).

NYTS is an annual cross-sectional, school-based, self-administered, pencil-and-paper questionnaire administered to U.S. middle and high school students.^†^ A three-stage cluster sampling procedure was used to generate a nationally representative sample of U.S. students attending public or private schools in grades 6–12. This report uses data from five NYTS waves (2014–2018). Sample sizes and response rates were 22,007 (73.3%) in 2014; 17,711 (63.4%) in 2015; 20,675 (71.6%) in 2016; 17,872 (68.1%) in 2017; and 20,189 (68.2%) in 2018.

Participants were asked about current (≥1 day during the past 30 days) use of cigarettes, e-cigarettes, hookahs, cigars, pipe tobacco, smokeless tobacco, snus, dissolvable tobacco products, and bidis. Current cigarette smoking was determined by asking “During the past 30 days, on how many days did you smoke cigarettes?” Current use of cigars was determined by asking “During the past 30 days, on how many days did you smoke cigars, cigarillos, or little cigars?” Current use of smokeless tobacco was determined by asking “During the past 30 days, on how many days did you use chewing tobacco, snuff, or dip?” Current use of e-cigarettes was determined by asking “During the last 30 days, on how many days did you use e-cigarettes?” Current use of hookahs was determined by asking “During the past 30 days, on how many days did you smoke tobacco in a hookah or waterpipe?” Current use of pipe tobacco (not hookahs), snus, dissolvable tobacco, and bidis were determined by asking “In the past 30 days, which of the following products have you used on at least one day?” “Any tobacco” use was defined as current use of one or more tobacco products. “Any smokeless tobacco” use was defined as current use of smokeless tobacco (chewing tobacco, snuff, or dip), snus, or dissolvables. Participants were also asked about any current use of tobacco products that were “flavored to taste like menthol (mint), alcohol (wine, cognac), candy, fruit, chocolate, or any other flavors.” Participants could select from a list of flavored tobacco products, including each noncigarette tobacco product type. Among students who reported current use of each product, those who selected the flavored product were categorized as flavored tobacco product users. Among current cigarette smokers, menthol smokers were categorized as those who reported “Yes” to the question “During the past 30 days, were the cigarettes that you usually smoked menthol,” or who reported “Newport” or “Kool” as the usual cigarette brand because these brands produce menthol cigarettes exclusively or predominantly.

Data were weighted to account for the complex survey design and adjusted for nonresponse; national prevalence estimates were calculated with 95% confidence intervals. Current flavored product use estimates for 2018 were assessed for any tobacco product and for each product individually, by school type, sex, and race/ethnicity. Use of flavored bidis was first included in the survey in 2016, so 2014–2015 data do not include bidis. For each school type, presence of linear and nonlinear (i.e., quadratic) trends were assessed during 2014–2018.^§^ For all analyses, p-values <0.05 were considered statistically significant. Analyses were conducted using SAS (version 9.4; SAS Institute) and SUDAAN (version 11.0.3; RTI International).

In 2018, 27.1% of high school students and 7.2% of middle school students reported current use of any tobacco product, corresponding to an estimated 4.92 million middle and high school students who use at least one tobacco product. Among current users of any tobacco product, 64.1% reported using at least one flavored tobacco product in the past 30 days. The percentage of current tobacco users who reported flavored product use in the past 30 days was 65.2% for e-cigarettes, 45.7% for menthol cigarettes, 43.6% for cigars, 38.9% for bidis, 37.5% for any smokeless tobacco, 26.5% for tobacco in pipes, and 26.1% for hookah (Table).

**TABLE:** Percentage of middle and high school students currently using tobacco products* who reported using flavored products^†^ during the preceding 30 days, by sex and race/ethnicity — National Youth Tobacco Survey, United States, 2018

Characteristic	Tobacco product % (95% CI)							
	Any tobacco^§^	E-cigarettes	Menthol cigarettes	Cigars	Any smokeless tobacco**	Hookahs	Pipe tobacco	Bidis
**Estimated no. of current tobacco product users^††^**	**4,920,000**	**3,640,000**	**1,410,000**	**1,310,000**	**1,100,000**	**740,000**	**200,000**	**130,000**
Estimated no. of flavored product users^††^	3,150,000	2,370,000	640,000	570,000	410,000	190,000	50,000	50,000
Prevalence of flavored product use among all students	11.7 (10.6–13.0)	9.0 (7.9–10.2)	2.5 (2.1–2.8)	2.2 (1.9–2.5)	1.6 (1.2–1.9)	0.7 (0.6–1.0)	0.2 (0.1–0.3)	0.2 (0.1–0.3)
Prevalence of flavored product use among current users	64.1 (61.6–66.6)	65.2 (62.6–67.8)	45.7 (42.1–49.4)	43.6 (40.1–47.2)	37.5 (33.0–42.2)	26.1 (21.5–31.2)	26.5 (19.7–34.6)	38.9 (28.7–50.2)
**School type**								
Middle school	48.7 (43.2–54.2)	51.5 (46.0–57.0)	42.0 (33.4–51.1)	39.4 (29.6–50.2)	28.4 (19.4–39.6)	18.3 (9.8–31.5)	—^§§^	—
High school	67.4 (64.8–70.0)	67.8 (65.0–70.4)	46.1 (41.9–50.3)	44.5 (40.8–48.3)	40.1 (35.3–45.1)	27.9 (22.7–33.7)	27.3 (18.9–37.6)	35.7 (24.1–49.4)
**Sex**								
Female	65.4 (62.3–68.4)	65.5 (62.4–68.5)	45.9 (39.7–52.2)	45.8 (40.1–51.6)	29.2 (22.5–36.9)	31.7 (24.5–39.8)	27.4 (16.8–41.4)	—
Male	63.1 (60.0–66.1)	62.5 (59.1–65.7)	45.4 (40.7–50.2)	42.4 (37.7–47.3)	41.2 (36.0–46.7)	20.5 (15.1–27.3)	25.5 (18.0–34.8)	26.3 (15.7–40.7)
**Race/Ethnicity**								
White, non-Hispanic	71.3 (68.3–74.0)	72.0 (69.0–74.7)	42.8 (37.9–47.8)	49.3 (44.3–54.4)	44.8 (39.3–50.5)	29.9 (21.2–40.4)	23.0 (13.8–35.8)	—
Black, non-Hispanic	46.5 (39.6–53.6)	52.8 (40.1–65.1)	51.4 (32.8–69.7)	39.1 (30.0–48.9)	—	—	—	—
Hispanic	54.3 (50.8–57.9)	49.3 (44.2–54.4)	50.6 (44.1–57.0)	39.2 (33.3–45.5)	22.1 (15.6–30.3)	26.7 (20.3–34.3)	37.0 (24.3–51.8)	—
Other, non-Hispanic	64.4 (57.5–70.8)	68.6 (62.0–74.5)	44.8 (35.0–54.9)	44.2 (30.7–58.5)	31.6 (19.2–47.3)	28.2 (17.3–42.5)	—	—

**Abbreviations:** CI = confidence interval; e-cigarettes = electronic cigarettes.

* Tobacco products asked about include cigarettes; e-cigarettes; hookahs (water pipes used to smoke tobacco); cigars (defined as cigars, cigarillos, or little cigars); tobacco in pipes; smokeless tobacco (defined as chewing tobacco, snuff, or dip); snus (a smokeless, spitless, tobacco product); dissolvable tobacco products (hereafter referred to as dissolvables); and bidis (small imported cigarettes wrapped in a leaf). Current cigarette smoking was determined by asking “During the past 30 days, on how many days did you smoke cigarettes?” Current use of cigars was determined by asking “During the past 30 days, on how many days did you smoke cigars, cigarillos, or little cigars?” Current use of smokeless tobacco was determined by asking “During the past 30 days, on how many days did you use chewing tobacco, snuff, or dip?” Current use of e-cigarettes was determined by asking “During the last 30 days, on how many days did you use e-cigarettes?” Current use of hookahs was determined by asking “During the past 30 days, on how many days did you smoke tobacco in a hookah or waterpipe?” Current use of pipe tobacco (not hookahs), snus, dissolvable tobacco, and bidis were determined by asking “In the past 30 days, which of the following products have you used on at least one day?”

^†^ Flavored tobacco product use was determined by the response to the question “Which of the following tobacco products that you used in the past 30 days were flavored to taste like menthol (mint), alcohol (wine, cognac), candy, fruit, chocolate, or other sweets?” Participants could select from a list of options to designate the flavored tobacco products they had used. Among those who reported any use of each respective product in the preceding 30 days, those who selected the flavored product were categorized as flavored product users; those who did not select the flavored product were categorized as only nonflavored product users; and those who did not provide any response to the flavored use question were assigned to missing flavor use status.

^§^ Any tobacco is use of cigarettes, cigars, smokeless tobacco, e-cigarettes, hookahs, pipe tobacco, snus, dissolvables, or bidis on ≥1 day in the preceding 30 days.

^¶^ Flavored cigarette use refers to menthol cigarettes. Menthol cigarette status was determined by asking “Menthol cigarettes are cigarettes that taste like mint. During the past 30 days, were the cigarettes that you usually smoked menthol?” and “During the past 30 days, what brand or cigarettes did you usually smoke?” Among past 30-day cigarette smokers, those responding “Yes” to the menthol question, or who reported “Newport” or “Kool” as the usual cigarette brand were classified as menthol smokers; subsequently those who reported “No” to the menthol question or who did not report “Newport” or “Kool” brands were classified as nonmenthol smokers; all other past 30-day cigarette smokers were classified as missing menthol smoking status.

** Any smokeless tobacco is current use of smokeless tobacco (chewing tobacco, snuff, or dip), snus, or dissolvables on ≥1 day in the preceding 30 days.

^††^ The estimated numbers of total and flavored tobacco product users were rounded down to the nearest 10,000.

^§§^ Dashes indicate data are statistically unreliable because the sample size was <50 or the relative standard error was >30%.

Among high school tobacco product users, a significant nonlinear decrease occurred during 2014–2018 in use of any flavored tobacco product (from 73.0% to 67.4%) (Figure). By product, a significant nonlinear increase in use of flavored e-cigarettes (from 65.1% to 67.8%) occurred; a significant nonlinear decrease in use of flavored smokeless tobacco (from 64.7% to 40.1%) occurred. Significant linear decreases in use of menthol cigarettes (from 54.5% to 46.1%), flavored hookah tobacco (from 63.8% to 27.9%), flavored cigars (from 64.7% to 44.5%), and flavored pipe tobacco (from 44.0% to 27.3%) occurred. Among middle school tobacco product users (Figure), a significant linear decrease in flavored hookah tobacco use (from 44.3% to 18.3%) occurred during 2014–2018. The use of flavored e-cigarettes decreased from 55.1% to 39.2% during 2014–2015 and then increased during 2015–2018 to 51.5%, comparable with the 2014 estimate. No significant change in use of any flavored tobacco, flavored smokeless tobacco, cigars, pipe tobacco, or menthol cigarettes among middle school students occurred during 2014–2018.

**FIGURE:**
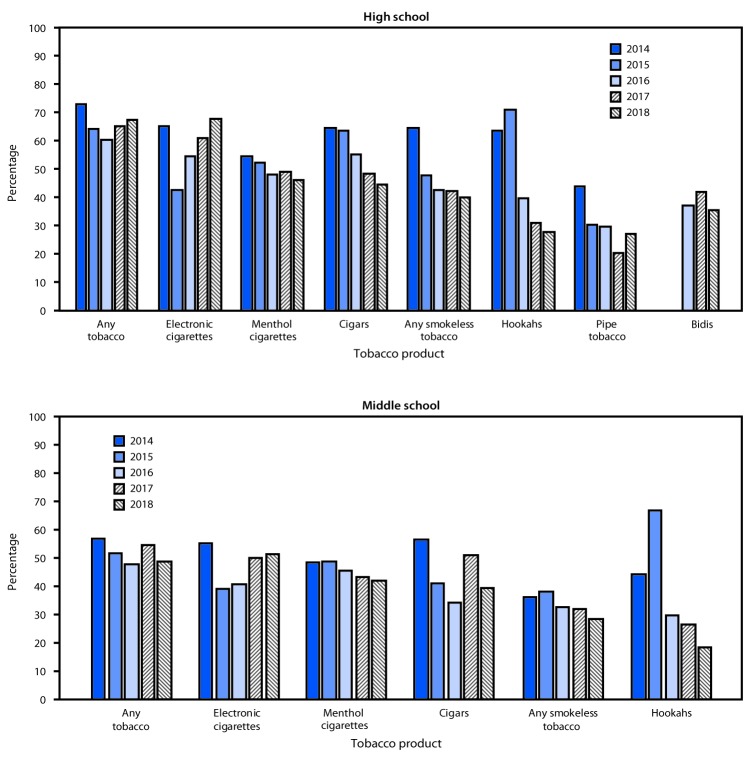
Percentage of current tobacco product*^,^^†^ users in high school and middle school who reported using flavored products during the preceding 30 days, by tobacco product — National Youth Tobacco Survey, United States, 2014–2018 * For 2014–2015, any tobacco is use of cigarettes, cigars, smokeless tobacco, e-cigarettes, hookahs, pipe tobacco, snus, or dissolvables on ≥1 day in the preceding 30 days. For 2016–2018, any tobacco is use of cigarettes, cigars, smokeless tobacco, e-cigarettes, hookahs, pipe tobacco, snus, dissolvables, or bidis on ≥1 day in the preceding 30 days. Exclusion of bidis from any tobacco use for 2016, 2017, and 2018 did not change the estimates. ^†^ Use of flavored bidis was only asked beginning in 2016, so estimates of flavored bidi use are not available for 2014–2015. For middle school estimates, use of flavored pipe tobacco and bidis are not shown because the individual estimates needed to be suppressed as a result of small sample size, relative standard error >30%, or both.

## Discussion

Nearly two thirds (3.15 million, 64.1%) of middle and high school student current tobacco product users reported current flavored tobacco product use in 2018. E-cigarettes were the most commonly used flavored tobacco product in 2018; flavored e-cigarette use has increased in recent years. During 2014–2018, use of any flavored tobacco product decreased among high school students who currently use tobacco products; however, no change occurred among middle school student users. The high prevalence of flavored tobacco product use among middle and high school students is a concern because flavors can increase the appeal of tobacco products to youths, promote youth initiation of tobacco products, and result in lifelong tobacco product use (*3*,*4*).

A recent examination of online tobacco retailers found that a sizable proportion of noncigarette tobacco products for sale in the United States are flavored (*5*). The recent increase in flavored e-cigarette use among youths might be due, in part, to the recent popularity and increased market share of e-cigarettes shaped like a USB flash drive, such as JUUL; these products can be used discreetly, have a higher nicotine content than earlier generation e-cigarettes, and are available in flavors that appeal to youths (*6*). These attributes might play a role in sustained use; research shows the majority of youths and young adults who reported ever using JUUL also reported being current JUUL users (*7*). Decreases in use of specific flavored tobacco products during the study period might be due to multiple factors, including actual decreases in flavored tobacco product use, a decrease in awareness that the product being used was flavored, or an increase in use of other products in hookahs (e.g., marijuana, herbal [nontobacco] products, or hashish) even though the survey question specifically refers to tobacco use in a hookah or waterpipe.

Population-based strategies at the state and local levels could help reduce use of flavored tobacco products by youths. In recent years, several communities have restricted the sale of flavored tobacco products. In 2009, New York City prohibited the sale of flavored cigars and smokeless tobacco products (excluding flavors such as menthol, mint, and wintergreen) except in adult-only tobacco bars^¶^. This law resulted in a significant decrease in cigar sales, compared with a 12% increase nationally during the same period (*8*). Providence, Rhode Island, passed a similar ordinance in 2012 prohibiting flavored tobacco product sales, including flavored e-cigarettes.** More recently, ordinances have also been adopted in Minneapolis, Minnesota; Oakland, California; San Francisco, California; Santa Clara County, California; St. Paul, Minnesota; and multiple municipalities in Massachusetts that include menthol among the types of prohibited flavors.^††^ In September 2019, Michgan became the first state to ban flavored e-cigarettes, including mint and menthol.^§§^ Continued evaluation of the impact of flavored tobacco product policies on tobacco-related behaviors is important, particularly among youths.

The findings in this report are subject to at least four limitations. First, data were collected from youths who attended public or private schools; therefore, the findings might not be generalizable to those who are home-schooled, have dropped out of school, or are in detention centers. Second, flavored tobacco product use was assessed using a check-all-that-apply response option, which might yield different estimates than forced-choice response options. Third, because of known underreporting of menthol cigarette smoking, this analysis relied on responses to menthol and usual brand questions, whereas determination of other flavored tobacco product use relied on a single question. Thus, results might not be directly comparable across products. Finally, NYTS only included use of bidis in the survey during 2016–2018.

On August 8, 2016, FDA finalized its deeming rule, which gave the agency jurisdiction over products made or derived from tobacco, including e-cigarettes, cigars, pipe tobacco, and hookah tobacco (*9*). On March 20, 2018, FDA released an advance notice of proposed rulemaking seeking public input on how best to regulate flavors, including menthol, in tobacco products.^¶¶^ In November 2018, FDA announced several new steps to protect youths, including restricting sales of flavored e-cigarettes (other than tobacco, menthol, mint, or nonflavored) to physical locations with age restrictions or online with heightened age verification procedures, and plans to publish advance notices of proposed rulemaking that would ban menthol cigarettes and cigars and all other flavored cigars.*** Further, FDA published draft guidance in March 2019 outlining a proposal to end the current compliance policy as it applies to flavored electronic nicotine delivery systems; a reprioritization of enforcement efforts will focus on mitigating risk for minors to access these tobacco products.^†††^ FDA regulation of the manufacturing, distribution, and marketing of flavored tobacco products, coupled with sustained implementation of comprehensive tobacco control and prevention strategies, can further reduce tobacco product initiation and use among youths (*2*,*10*).

**Summary**

**What is already known about this topic?**

Flavored tobacco products can appeal to youths and young adults.

**What is added by this report?**

During 2014–2018, current use of flavored electronic cigarettes increased among high school students and during 2015–2018 among middle school students. During 2014–2018, current use of flavored hookah tobacco decreased among middle and high school students. Current use of other flavored tobacco products decreased among high school students but did not change among middle school students.

**What are the implications for public health practice?**

Food and Drug Administration regulation of the manufacturing, distribution, and marketing of flavored tobacco products, coupled with sustained implementation of comprehensive tobacco control and prevention strategies, can further reduce tobacco product use among youths.

## Footnotes

* Family Smoking Prevention and Tobacco Control Act [Pub. L. No. 111–31, H.R. 1256 (2009)]. https://www.govinfo.gov/content/pkg/PLAW-111publ31/html/PLAW-111publ31.htm.

† https://www.cdc.gov/tobacco/data_statistics/surveys/nyts/index.htm.

§ A test for linear trend is significant if an overall statistically significant decrease or increase occurs during the study period. Data were also assessed for the presence of nonlinear (i.e., quadratic) trends; a significant nonlinear (i.e., quadratic) trend indicates that the rate of change accelerated or decelerated across the study period.

¶ https://www.lawserver.com/law/state/new-york/ny-laws/ny_new_york_city_administrative_code_17-715.

** https://www.publichealthlawcenter.org/sites/default/files/resources/US-Sales-Restrictions-Flavored-Tobacco-Products-2017.pdf.

†† https://library.municode.com/mn/minneapolis/codes/code_of_ordinances?nodeId=COOR_TIT13LIBURE_CH281TODE_281.15DE; https://www.eastbaytimes.com/2017/09/20/oakland-bans-flavored-tobacco-products/; https://sfgov.legistar.com/View.ashx?M=F&ID=5274235&GUID=86C18253-BA63-4C0F-A6A0-E881211D2CB7; https://library.municode.com/ca/santa_clara_county/ordinances/code_of_ordinances?nodeId=796084; https://stpaul.legistar.com/LegislationDetail.aspx?ID=3145418&GUID=42F0956E-EBB2-43E4-A74C-76FCC9A9E37C&FullText=1; http://mhoa.com/wp-content/uploads/2018/12/muni-list-Flavored-OTP-Restriction.pdf.

§§ https://www.washingtonpost.com/health/michigan-becomes-first-state-to-ban-flavored-e-cigarettes/2019/09/03/34f234c6-ce4c-11e9-8c1c-7c8ee785b855_story.html.

¶¶ https://www.federalregister.gov/documents/2018/03/21/2018-05655/regulation-of-flavors-in-tobacco-products.

*** https://www.fda.gov/news-events/press-announcements/statement-fda-commissioner-scott-gottlieb-md-proposed-new-steps-protect-youth-preventing-access.

††† https://www.fda.gov/regulatory-information/search-fda-guidance-documents/modifications-compliance-policy-certain-deemed-tobacco-products.
